# Loss of Mgat5a-mediated *N*-glycosylation stimulates regeneration in zebrafish

**DOI:** 10.1186/s13619-016-0031-5

**Published:** 2016-10-20

**Authors:** Wuhong Pei, Sunny C. Huang, Lisha Xu, Kade Pettie, María Laura Ceci, Mario Sánchez, Miguel L. Allende, Shawn M. Burgess

**Affiliations:** 1 Functional and Translation Genome Branch, National Human Genome Research Institute, 9000 Rockville Pike, Building 50, Room 5537, Bethesda, MD 20892 USA; 2 Center for Genome Regulation, Facultad de Ciencias, Universidad de Chile, Casilla 653, Santiago, Chile

**Keywords:** Zebrafish, CRISPR/Cas9, mgat5a, *N*-glycosylation, Regeneration

## Abstract

**Background:**

We are using genetics to identify genes specifically involved in hearing regeneration. In a large-scale genetic screening, we identified *mgat5a*, a gene in the *N*-glycosylation biosynthesis pathway whose activity negatively impacts hair cell regeneration.

**Methods:**

We used a combination of mutant analysis in zebrafish and a hair cell regeneration assay to phenotype the loss of Mgat5a activity in zebrafish. We used pharmacological inhibition of *N*-glycosylation by swansonine. We also used over-expression analysis by mRNA injections to demonstrate how changes in *N*-glycosylation can alter cell signaling.

**Results:**

We found that *mgat5a* was expressed in multiple tissues during zebrafish embryo development, particularly enriched in neural tissues including the brain, retina, and lateral line neuromasts. An *mgat5a* insertional mutation and a CRISPR/Cas9-generated truncation mutation both caused an enhancement of hair cell regeneration which could be phenocopied by pharmacological inhibition with swansonine. In addition to hair cell regeneration, inhibition of the *N*-glycosylation pathway also enhanced the regeneration of lateral line axon and caudal fins. Further analysis showed that *N*-glycosylation altered the responsiveness of TGF-beta signaling.

**Conclusions:**

The findings from this study provide experimental evidence for the involvement of *N*-glycosylation in tissue regeneration and cell signaling.

**Electronic supplementary material:**

The online version of this article (doi:10.1186/s13619-016-0031-5) contains supplementary material, which is available to authorized users.

## Background

Glycans, together with nucleic acids, proteins, and lipids, constitute the four major components of the cell [1]. Glycans have great structural complexity and are largely diversified in different cell types providing cells with distinct functional properties [2]. Due to technical challenges in the detection and characterization of different sugar forms, glycan research has generally lagged behind genomic and proteomic approaches. The biological consequences of altered glycosylation remains a critical but challenging field of biomedical research [3].

Glycosylated proteins represent a prominent form of glycan conjugates in the cell. Glycans can be found on a wide variety of proteins, ranging from secreted extracellular proteins, to membrane receptors, to cytosolic proteins [4]. Glycosylation on these proteins offers a potential for modulation of intracellular signaling and intercellular communications [5, 6]. There are two major types of protein glycosylation, with *N*-glycosylation adding sugars to the nitrogen of asparagine and *O*-glycosylation adding sugars to the oxygen of serine or threonine residues [7]. It is evident that one single protein can be glycosylated at multiple residues by one or multiple forms of glycosylation. However, the dynamics and the impact of specific glycosylation events are largely unknown, partly due to lack of adequate knowledge on the glycosylation processing enzymes themselves.

Tissue regeneration after traumatic injury is a process demanding complex orchestration of molecular signaling and cell-cell interactions. Since mammals have relatively limited regeneration potential, many non-mammalian vertebrate models, including zebrafish, have been used to study the molecular mechanisms behind regeneration of tissues such as heart, liver, and hair cells (hearing) [8, 9]. These studies have demonstrated a critical involvement of numerous proteins and associated signaling pathways during tissue regeneration; however, the contribution of protein glycosylation in this process remains under-investigated.

In a genetic screen for genes affecting hair cell regeneration in zebrafish, we identified *mgat5a* as a modifier in the regenerative response. The *mgat5a* gene is highly conserved across all vertebrates. It encodes for the beta1,6-*N*-acetylglucosaminyl-transferase V enzyme, a medial golgi enzyme in *N*-glycosylation pathway catalyzing the biosynthesis of b1,6-GlcNac-branched *N*-glycosylation to various protein conjugates. Previous reports indicate that Mgat5a plays an important role in tumor metastasis, cell proliferation, and immune cell activation [10–14]. In this study, we examined the spatio-temperal distribution of *mgat5a* transcript during embryo development and explored the function of Mgat5a and the *N*-glycosylation pathway in injury-induced tissue regeneration.

## Results

### An Mgat5a-RFP insertional mutation enhances hair cell regeneration

**Fig. 1:**
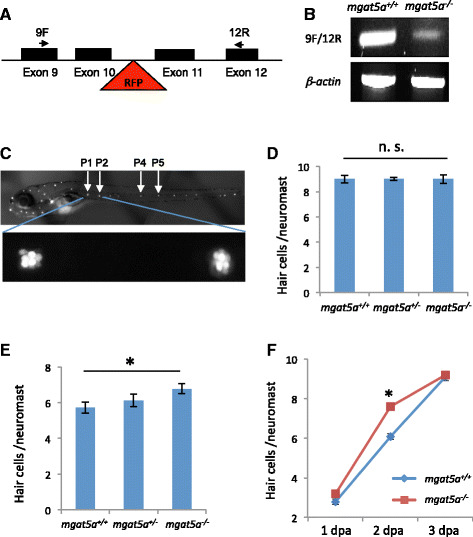
*mgat5a* ^*mn0157gt*^ mutation enhances hair cell regeneration. **a** Schematic diagram of the gene-trap mutation in the *mgat5a* gene. Exons are indicated by *solid boxes*. The *red triangle* indicates the site of the gene trap insertion. *Arrows* indicate the locations of primers used for mutation detection. **b** RT-PCR analysis of *mgat5a* expression in the wild-type (WT) and homozygous mutant embryos. B-actin is used as internal reference. **c** Fluorescent images of a wild-type embryo at 5 dpf stained with Yo-Pro-1. *Upper pattern* shows the distribution of neuromasts. The neuromasts used for hair cell counting are labeled. *Bottom panel* shows a higher magnification of P1 and P2 neuromasts. **d** Normal hair cell development in *mgat5a* ^*mn0157gt*^ mutants. **e** Enhanced hair cell regeneration in *mgat5a* ^*mn0157gt*^ mutants. **f** Time-course analysis for hair cell regeneration in *mgat5a* ^*mn0157gt*^ mutants. Graphs **d**, **e**, and **f** show the data from analyzing 24–32 embryos that were generated from a pairwise incross of heterozygotes and genotyped afterwards for genotype-phenotype correlation. *Error bars* show the s.e.m. The difference between wild type (WT) and homozygotes is not significant (n. s.) in **d** (*p* = 0.83), but significant in **e** (*p* = 0.02) and in **f** at 2 dpa (*p* < 0.001)

To identify key genes and pathways involved in hearing regeneration, we performed a large-scale targeted mutagenesis and genetic screen to identify genes essential for the regeneration of hair cells in the zebrafish lateral line (Pei and Burgess, unpublished data). We specifically targeted a subset of genes selected from previous work where we performed transcriptional profiling on regenerating sensory epithelia of adult zebrafish after damaging the inner ear with intense sound exposure [15]. Genes were targeted using clustered regularly interspaced short palindromic repeats (CRISPR)-associated protein 9 (Cas9) or DNA integration and then inbred to homozygosity to test for hair cell regeneration after exposure to CuSO_4_ for 1 h. Hair cells were counted 48 h after toxic exposure. We identified *mgat5a* as a modifier of the regenerative response based on analyzing *mgat5a* ^*mn0157gt*^ mutation. *mgat5a* ^*mn0157gt*^ was created by inserting a gene-trap transposon into intron 10 of the *mgat5a* gene and thus producing a fusion protein with a truncated Mgat5a protein at the N terminus and red fluorescent protein (RFP) at the C terminus [16]. Reverse-transcription PCR (RT-PCR) analysis with two primers framing the gene-trap integration region revealed that the mutant had a dramatically reduced but residual expression of *mgat5a* wild-type mRNA (Fig. 1a,b), indicating that *mgat5a* ^*mn0157gt*^ is a hypomorphic allele. The *mgat5a* ^*mn0157gt*^ mutation had no effect on hair cell development (Fig. 1c,d); however, it possessed a measurable increase in hair cell regeneration compared to wild-type (Fig. 1e). A time-course analysis revealed that hair cell regeneration in the mutant was enhanced at 2 days post ablation (dpa), but became comparable to the control at 3 dpa, indicating that it was not an “overgrowth” phenotype (Fig. 1f). No additional phenotypes were observed in the *mgat5a* ^*mn0157gt*^ mutant.

### *mgat5a* is expressed in neuromast hair cells and supporting cells, but not in mantle cells

**Fig. 2:**
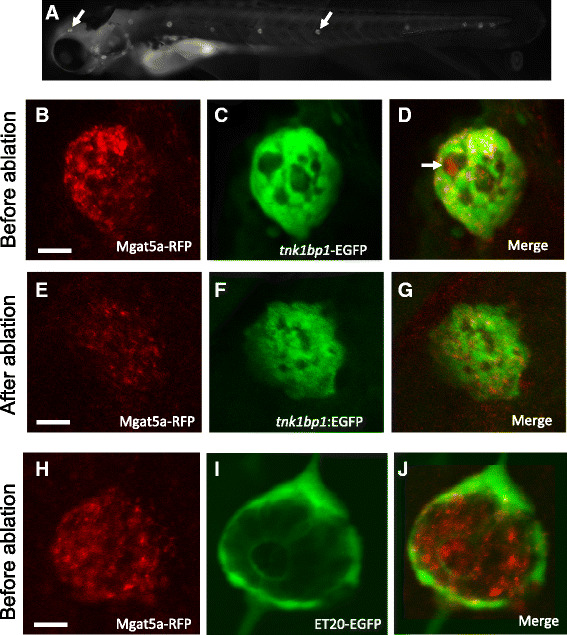
*mgat5a* is expressed in neuromast hair cells and supporting cells, but not in mantle cells. **a** RFP expression in *mgat5a* ^*mn0157gt*^ mutant. *mgat5a* ^*mn0157gt*^ homozygous embryos at 5 dpf were used for the RFP expression analysis. *Arrows* point to the RFP expression in the neuromasts in the head and trunk. **b–g** *mgat5a* is expressed in hair cells and supporting cells, revealed by the supporting cell labeling transgenic line Et (*tnk1bp1*:EGFP). When compared to the neuromasts before ablation (**b–d**), the neuromasts after hair cell ablation (**e** and **g**) display a dramatic reduction in RFP level. *White arrows* in **d** points to the expression of RFP in hair cells. Granulated distribution of *yellow* color in **d** indicate the co-localization of RFP and GFP in supporting cells. **h–j** *mgat5a* is not expressed in mantle cells, revealed by mantle cell labeling transgenic line Et (*ET20*:*ET20:EGFP*). RFP and GFP are localized in different areas of the neuromasts. The columns from *left* to *right* are the images from *red*, *green*, and merge channels. *Scale bars* 10 μm

By visualizing the RFP in the *mgat5a* ^*mn0157gt*^ mutant, we examined the localization of *mgat5a* in zebrafish embryos. Microscopic examination revealed that the RFP was present in both anterior and lateral line neuromasts (Fig. 2a), another hair cell-containing organ in zebrafish [17]. Confocal analysis revealed that the Mgat5a-RFP fusion protein was expressed through the entire neuromast in a somewhat granulated pattern (Fig. 2b, h). The granulated distribution is consistent with the Golgi localization of wild-type Mgat5a protein.

To identify which cell types in neuromast were expressing *mgat5a*, we first crossed the *mgat5a* ^*mn0157gt*^ line with Et (*tnk1bp1*:EGFP), in which the *tnk1bp1* promoter drives the expression of a green fluorescent protein (GFP) specifically in supporting cells (Fig. 2c) [18]. The resulted double transgenic embryos displayed granulated co-localized fluorescence in some area of the neuromast (Fig. 2d), indicating expression of GFP and RFP in supporting cells. The double transgenic embryos also displayed granulated RFP in some GFP-excluded areas, indicating *mgat5a* expression in hair cells (Fig. 2d). It is interesting to note that most of the RFP expression did not usually directly co-localize with GFP, even when expressed in the same cells. We believe that this is because the Mgat5a enzyme is sequestered in the golgi apparatus and the GFP expression is cytoplasmic. To further verify the presence of *Mgat5a* in hair cells, we ablated hair cells using ototoxic chemical copper and then examined the expression levels of the GFP and RFP. After copper exposure, all cells expressed both GFP and RFP, indicating that the GFP-negative, RFP-positive cells were the hair cells.

We also crossed *mgat5a* ^*mn0157gt*^ with the transgenic line Et (ET20: EGFP), in which the GFP specifically labels the neuromast mantle cells (Fig. 2i) [19]. We found the resulting double transgenic embryos allocated the RFP and GFP proteins in different areas of neuromast, with *Mgat5a*-RFP in the inner circle of the neuromast (supporting cells) and ET20: EGFP predominantly at the periphery of the neuromast (mantle cells), and no significant overlap between these two fluorescent proteins (Fig. 2j). This distribution pattern indicates that *mgat5a* is not expressed in the mantle cells.

### *mgat5a* is expressed in neurological tissues during zebrafish embryo development

**Fig. 3:**
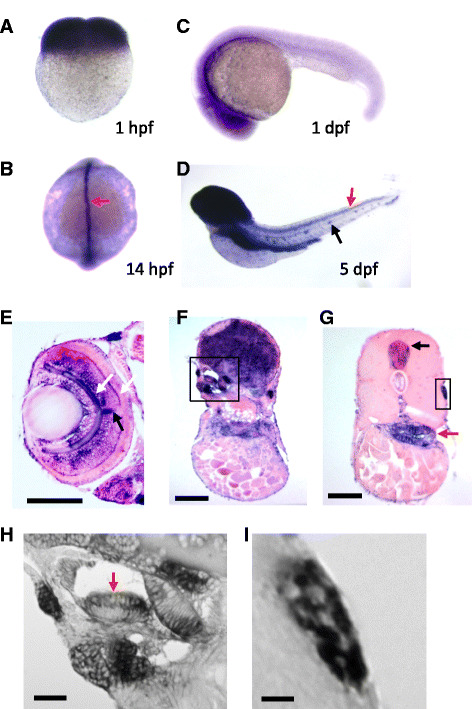
*mgat5a* expression during zebrafish embryo development. **a–d** Whole mount in situ hybridization analysis of *mgat5a* expression at 1 hpf (**a**), 14 hpf (**b**), 1 dpf (**c**), and 5 dpf (**d**). Orientations: lateral view in **a**, **c,** and **d**; dorsal view in **b**. *Red arrow* in **b** points to the expression in the dorsal midline. *Red* and *black* arrows in **d** point to the expression in spinal cord and lateral line neuromast. **e–i** Histology sectioning analysis of *mgat5a* expression in the retina (**e**), brain (**f**), and trunk (**g**). *White arrows* in **e** point to the expression in outer and inner plexiform layers of the retina. Black arrow in e points to the expression in the optic nerve. *Black box* in **f** demarcates the inner ear area. *Black* and *red arrows* in **g** point to the expression in the spinal cord and gut, respectively. *Black box* in **g** demarcates lateral line neuromast. **h** A magnified image of the inner ear region demarcated in **f**. *Red arrow* points to the enriched expression in the apical region of cristae. **i** A magnified image of the neuromast demarcated in **g**. Mosaic patterning indicates expression in some neuromast cells. *Scale bars* 100 μm in **e**, **f**, and **g**; 20 μm in **h** and **i**

The *mgat5a* gene is conserved across vertebrates including zebrafish, mice, and humans. To study the function of *mgat5a* during zebrafish embryo development, we performed whole-mount in situ hybridization (WISH) analysis to examine the spatio-temperal localization of *mgat5a* transcripts in embryonic tissues. We found that *mgat5a* was strongly expressed at the four-cell stage (Fig. 3a), prior to the onset of zygotic transcription, indicating that the transcript was maternally deposited. There was an enrichment of *mgat5a* expression in the dorsal midline at 14 h post fertilization (hpf) (Fig. 3b) and broadly in the brain at 1 dpf (Fig. 3c). The brain expression persisted at 5 dpf and expression was also detected in the gut, spinal cord, and lateral line neuromasts (Fig. 3d).

Histological sections of in situ-stained 5-day-old embryos revealed increased expression of *mgat5a* in several organs. It was highly expressed in many retinal tissues including the optic nerve and the inner and outer plexiform layers (Fig. 3e). Sections revealed the presence of high levels of *mgat5a* transcripts in the entire brain area (Fig. 3f), the cristae of the inner ear (Fig. 3f,h), and the spinal cord (Fig. 3g). Histological section analysis also revealed an uneven distribution of *mgat5a* expression in neuromasts (Fig. 3g,I), supporting its expression in a subpopulation of neuromast cells. In addition, the enrichment of *mgat5a* was detected in the gut (Fig. 3g), an organ known for high activity of glycosylation enzymes [20].

### An *mgat5a* deletion mutation enhances hair cell regeneration

**Fig. 4:**
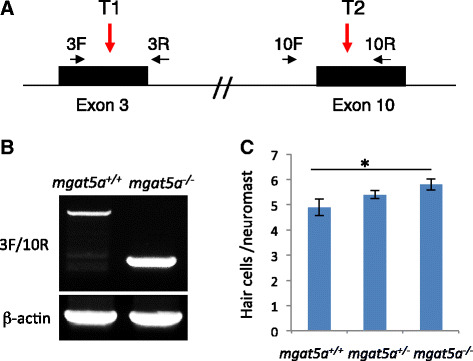
*mgat5a* ^*hg29*^ mutation enhances hair cell regeneration. **a** Schematic diagram of the *mgat5a* ^*hg29*^ mutation. Locations of the two CRISPR targets are indicated by *red arrows*. The primers used for mutation detection and knockdown analysis are indicated by *black arrows*. The resulted *mgat5a* E3-E10 mutation deletes 49.2 kb of genomic DNA between the T1 and T2 CRISPR targets. **b** RT-PCR analysis of *mgat5a* expression in wild-type (WT) and homozygous mutant embryos. E3F/E10R primer pair produces an amplicon of 985 bp in the WT embryos and an amplicon of 244 bp in the mutant embryos. Beta-actin is used as internal reference. **c** Enhanced hair cell regeneration in *mgat5a* ^*hg29*^ mutants. The graph is obtained from analyzing and afterwards genotyping 32 embryos that were generated from a pairwise incross of heterozygotes. *Error bars* show the s.e.m.. The difference between wild-type (WT) and homozygotes in **d** is significant (*p* = 0.02)

To verify the role of *mgat5a* in hair cell regeneration, we used CRISPR/Cas9 mutagenesis to create insertion/deletion (indel) mutations in the *mgat5a* gene (Fig. 4a). Small indel mutations in either target 1 (T1) in exon 3 or target 2 (T2) in exon 10 caused no effects on hair cell development or regeneration (data not shown). When injecting both single guide RNA targets simultaneously, we created a deletion mutation (*mgat5a* ^*hg29*^) that deleted 49 kb of genomic DNA spanning from exon 3 to the exon 10 of the 17-exon *mgat5a* gene. RT-PCR analysis revealed that the deletion mutant produced a truncated *mgat5a* mRNA, whose expression level was higher than that of the wild-type *mgat5a* mRNA in the control sibling (Fig. 4b). The deletion mutant showed no effect on hair cell development (data not shown), but like the gene-trap allele *mgat5a* ^*mn0157gt*^, showed an enhancement of hair cell regeneration that was detectable 2 dpa (Fig. 4c). Similar to *mgat5a* ^*mn0157gt*^, the *mgat5a* ^*hg29*^ mutant showed no obvious abnormalities and lived as a generally healthy and fertile adult.

### CRISPR mutations of other genes in the *N-*glycosylation pathway had no effect on hair cell regeneration

Mgat5a is a component in *N*-glycosylation pathway. To examine the role of other components in the *N*-glycosylation pathway in hair cell regeneration, we used CRISPR/Cas9 targeting to mutate 11 other genes that function at different steps of *N*-glycosylation pathway (Additional file 1: Figure S1, Additional file 2: Table S1). We found none of the mutated genes as single mutations had an effect on hair cell regeneration (data not shown). We also found no alteration in hair cell regeneration in mutated *mgat5b* (data not shown), a gene that has presumably overlapping function with *mgat5a* and is conserved across many species [21]. The failure in identifying other gene candidate may suggest that many components in *N*-glycosylation pathway have functional redundancy and/or a strong compensatory response.

### Inhibition of *N*-glycosylation pathway enhances hair cell regeneration

**Fig. 5:**
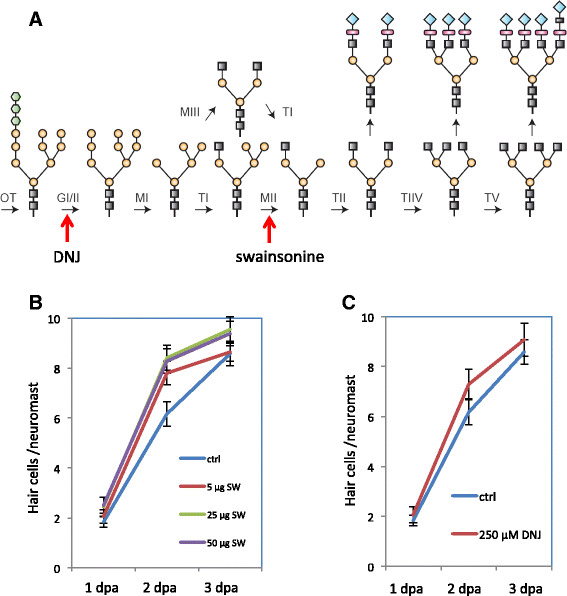
Inhibition of the *N*-glycosylation pathway enhances hair cell regeneration. **a** The *N*-glycosylation pathway and the inhibition targets of swainsonine and 1-deoxynojirimycin (DNJ). The targets of inhibition for swainsonine and DNJ are indicated by *red arrows*. **b** Swainsonine treatment causes a transient increase in hair cell regeneration. There is a significant difference at 2 dpa between the control and 5 μg/ml swainsonine treatment, as well as between the control and 50 μg/ml of swainsonine (*p* < 0.001 for both). There is no difference between swainsonine at 5 and 50 μg/ml (*p* = 0.063). **c** DNJ treatment causes a transient increase in hair cell regeneration. *Graphs* represent data from the analysis of 10 embryos per data point. *Error bars* show the s.e.m. There is a significant difference at 2 dpa between the control and 250 μM of DNJ (*p* = 0.014)

Both the *mgat5a* ^*mn0157gt*^ insertion mutant and the *mgat5a* ^*hg29*^ deletion mutant had considerable mRNA expression in either wild-type form (Fig. 1b) or truncated form (Fig. 4b), suggesting both mutations are hypomorphic. To examine the impact of loss-of-function of *N*-glycosylation pathway on hair cell regeneration, we used two pharmacological inhibitors, swainsonine (SW) and 1-deoxynojirimycin (DNJ), targeting different steps of the pathway (Fig. 5a). Both inhibitors caused enhanced hair cell regeneration at 2 dpa and no difference at 3 dpa (Fig. 5b,c), consistent with the observations from *mgat5a* ^*mn0157gt*^ and *mgat5a* ^*hg29*^ deletion mutants. DNJ had a poor solubility, the only concentration that showed a significant effect was 250 μM. SW enhanced hair cell regeneration at several concentrations. SW at 5 μg/ml significantly enhanced hair cell regeneration; however, increased concentration of SW (at 25 or 50 μg/ml) did not exaggerate the difference, suggesting a complete inhibition of the pathway with 5 μg/ml of SW. Since SW had a stronger effect on hair cell regeneration than DNJ and the two *mgat5a* hypomorphic mutations, it was used for further investigation on the role of *N*-glycosylation in other tissue regeneration.

### Inhibition of *N*-glycosylation pathway enhances the regeneration of lateral line axon and caudal fin

**Fig. 6:**
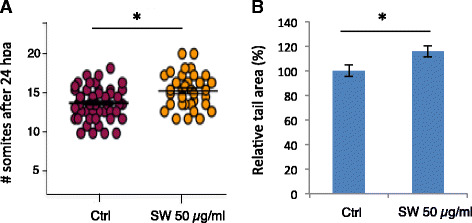
Inhibition of the *N*-glycosylation pathway enhances regeneration of the lateral line axon (**a**) and caudal fin (**b**). For lateral line axon regeneration, 3 dpf embryos had their lateral line neuron neurectomized and allowed to recover for 24 h. Somite position for the lead edge of the regenerating neuron was then determined. For caudal fin regeneration, WT embryos were amputated at 3 dpf, treated without or with SW for 72 h, and then the area of caudal fin regeneration was measured. *Graphs* show the mean and s.e.m. A significant difference is detected between the control and 50 μg/ml of swainsonine in axon regeneration (*n* = 45 for control, *n* = 33 for SW, *p* = 0.0015) and fin regeneration (*n* = 21, *p* = 0.029)

Since *mgat5a* is expressed in multiple tissues (Fig. 3) and *N*-glycosylation affects numerous biological processes [1], we hypothesized that blocking *N*-glycosylation would affect different forms of regeneration. We first examined the effect of SW on lateral line axon regeneration. The lateral line axon is an essential part of the peripheral nerve system in zebrafish that innervates the lateral line neuromasts [17]. In addition to hair cell regeneration, we found that SW treatment significantly promoted the regeneration of laser-damaged lateral line axons (Fig. 6a). We also examined the effect of SW on the regeneration of caudal fins, a process involving the regrowth of multiple tissue types, and found that SW significantly promoted caudal fin regeneration (Fig. 6b).

### Alterations in Notch signaling do not explain the observed phenotypes

**Fig. 7:**
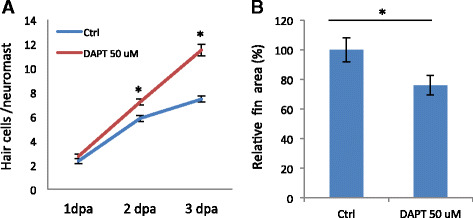
Inhibition of Notch signaling causes differences in regeneration compared to inhibition of *N*-glycosylation. **a** DAPT treatment leads to a continuous addition of hair cells during regeneration unlike swansonine that levels off after 2 dpa. A significant difference is detected at 2 dpa (*n* = 10, *p* < 0.001) and 3 dpa (*n* = 10, *p* < 0.001), but not at 1 dpa (*n* = 10, *p* = 0.132), between the control and DAPT-treated embryos. **b** DAPT treatment inhibits caudal fin regeneration. The difference between untreated and DAPT treated fish is significant (*n* = 10, *p* = 0.026)

We hypothesized that because the majority of glycosylated proteins are at the cell surface, altering glycosylation patterns could impact the dynamics of cell signaling. The Notch signaling pathway is a candidate, as several components of the Notch pathway are extensively *N*-glycosylated [22, 23]. In addition, Notch signaling is one of the few known pathways that can promote hair cell regeneration [8]. To study whether SW impacts Notch signaling during hair cell regeneration, we compared the effect of SW and Notch signaling inhibitor *N*-[(3,5-difluorophenyl)acetyl-L-alanyl-2-phenyl] glycine-1,1-dimethylethyl ester (DAPT) and determined their effects on tissue regeneration. For hair cell regeneration, DAPT treatment enhanced hair cell regeneration at 2 dpa, similar to SW. The DAPT-mediated enhancement persisted and hair cell numbers were higher than the controls at 3 dpa (Fig. 7a), consistent with the previous report [24]. However, the SW-mediated regeneration was statistically the same as the controls at 3 dpa (Fig. 5b). In caudal fin regeneration (Fig. 6b), DAPT treatment had the opposite effect of SW causing an inhibition of regeneration (Fig. 7b).

### Inhibition of *N*-glycosylation pathway reduces responsiveness to TGF-beta antagonists but not agonists

**Fig. 8:**
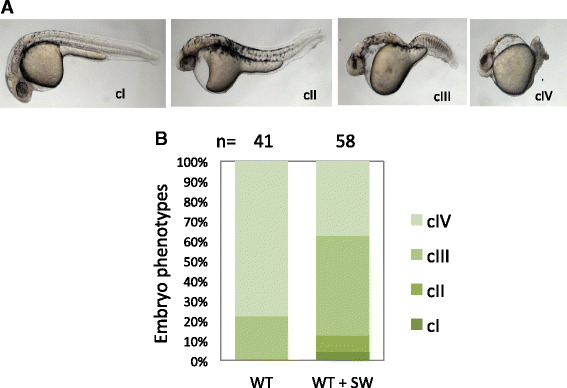
Inhibition of *N*-glycosylation reduces responsiveness to TGF-beta antagonist Lefty1. **a** Classification of morphological phenotypes at 1 dpf. WT embryos were injected with 25 pg of *lefty1* mRNA at one-cell stage, treated without or with 50 μg/ml SW from 4 hpf until 24 hpf, and then used for phenotypic analysis. Representative phenotypes for each class of embryos are shown, with the embryos oriented in a lateral view. cI–cIV, class I–class IV. **b** Percentage of embryos with different classes of phenotypes. Number of embryos analyzed is as indicated. A significant difference is detected between the control and SW-treated groups (*p* < 0.001)

Several in vitro studies have shown that *N*-glycosylation affects TGF-beta signaling by altering the responsiveness to TGF-beta ligands [12, 25–27]. To test whether SW affects tissue regeneration in vivo by altering TGF-beta signaling, we examined the morphological phenotypes of the embryos injected with synthetic mRNA of two TGF-beta/Nodal ligands: Lefty1 and Squint [28, 29], in the presence or absence of SW. The presence of SW significantly reduced the responsiveness of the embryos to the mRNA encoding Lefty1, a Nodal antagonist (Fig. 8a,b). However, SW showed no significant effect on the embryos injected with the mRNA encoding Squint, a Nodal agonist (Additional file 1: Figure S2). These data indicate that the SW-treated embryos are more resistant to a reduction in Nodal signaling, suggesting a potential up-regulation of TGF-beta signaling in the SW-treated embryos.

We have not specifically identified members of the TGF-beta superfamily whose alterations result in the observed phenotypes in the *mgat5a* mutants or swansonine-treated embryos, but it remains an area of active research.

## Discussion

Several features of zebrafish make it a unique model for large-scale genetic screening, including large egg production, a completed genome sequence, a flexible genome-editing toolset, and relatively low cost compared to other vertebrate models. Using zebrafish to study the regeneration of the tissues that do not typically regenerate in mammals allows us to discover key pathways necessary to trigger wound healing.

Our screening reveals that both the *mgat5a* ^*mn0157gt*^ gene trap mutant and the *mgat5a* ^*hg29*^ deletion mutant caused a transient enhancement of hair cell regeneration. The detectable level of wild-type *mgat5a* mRNA in the *mgat5a* ^*mn0157gt*^ mutant is likely from some skipping of the gene trap splice acceptor. The increased level of truncated mRNA in the *mgat5a* ^*hg29*^ deletion mutant could be caused by a feedback compensation for reduced Mgat5a enzymatic activity. Our sequencing analysis indicated the truncated mRNA contains a frame-shifted mutation that presumably encodes an extra 23 residues followed by a stop codon (data not shown). Since the deletion region spans 8 exons in the middle of the 17-exon gene, it is possible there is an alternative splicing of the truncated mRNA to produce a partially functional Mgat5a protein. We have not yet detected any alternative transcripts.

WISH analysis demonstrated an enrichment of *mgat5a* in neurological tissues including the brain and retina during early larval development. In addition, *mgat5a* is highly expressed in hair cells and supporting cells, the neurosensory receptor and glia-like cells that are responsible for detecting sound and motion. This expression pattern is consistent with the role of *N*-glycosylation in nerve development [30]. Hair cell regeneration was measurably accelerated in the *mgat5a* ^*mn0157gt*^ mutant and the *mgat5a* ^*hg29*^ deletion mutant, a phenotype that could be recapitulated by SW inhibition of the *N*-glycosylation pathway, suggesting that *N*-glycosylation also plays a role in nerve regeneration. This hypothesis was further supported by the observation that SW inhibition enhanced regeneration of the lateral line axons that innervate hair cells. It is possible that the enhanced regeneration of caudal fins could also be, at least partially, attributed to the enhanced nerve regeneration in the fin tissue, as nerve signaling has been shown to be important for limb regeneration in axolotls [31].

It is challenging to identify which signaling pathways are affected by Mgat5a-mediated *N*-glycosylation during tissue regeneration due to its involvement in a wide variety of cellular functions. *N*-glycosylation modifies numerous proteins and can affect protein functionality through different mechanisms [32]. Previous studies have shown that Mgat5a has a variety of functions during growth and homeostasis. Mgat5a is important for tumor growth and metastasis and *mgat5a* mutant showed reduced tumor cell mobility [11, 27, 33, 34]. Mgat5a plays a role in immunity, as Mgat5a deficiency is associated with abnormal T cell activation and autoimmunity [10, 13]. Up-regulation of Mgat5a facilitates keratinocyte proliferation, epidermal hyperplasia, and skin wound healing [12, 14]. In addition, a secreted version of Mgat5a promotes angiogenesis [35]. Furthermore, Mgat5a can modulate the functions of different cytokine receptors [27].

We probed the involvement of Notch signaling pathway because its components are *N*-glycosylated and it plays a role in promoting hair cell regeneration [22–24, 36], by comparing the effect of *N*-glycosylation inhibition with SW and Notch signaling inhibition with DAPT. For hair cell regeneration, the effects of SW and DAPT were similar at 2 dpa but different at 3 dpa. SW promoted axon regeneration, consistent with the effects of DAPT [37]. For caudal fin regeneration, the effect of SW is opposite to that of DAPT [38, 39]. Together, these data suggest that SW-mediated caudal fin regeneration occurs in a Notch-independent mechanism, while SW-mediated regeneration of hair cell and axon may depend on Notch signaling activation. This possibility requires further examination.

TGF-beta signaling is one of the prominent pathways affected by *N*-glycosylation based on in vitro studies [12, 26, 27]. We found that inhibition of TGF-beta signaling inhibited the regeneration of hair cells and caudal fins (data not shown), the opposite of the effects of SW. Introduction of embryonic TGF-beta/Nodal ligands revealed a reduced responsiveness to the antagonist Lefty1 but not to the agonist Squint, suggesting a possible up-regulation of TGF-beta signaling in the SW-treated embryos. Based on the diversified functions of Mgat5a, TGF-beta signaling is not likely to be the sole contributor for the *N*-glycosylation effects seen in tissue regeneration. To make it more complicated, the effects on TGF-beta signaling could be due to cross-talk with Notch signaling [40, 41]. Much more work is needed to elucidate why the loss of Mgat5a-mediated *N*-glycosylation results in an improved regeneration.

## Conclusions

Our study demonstrates a role for Mgat5a in modulating the regeneration of multiple tissues, proposing *N*-glycosylation as a potential therapeutic target for tissue regeneration. We also show that alterations in glycosylation can alter the sensitivity of cell signaling in certain pathways.

## Methods

### Zebrafish embryology

All animal work was in compliance with NHGRI IACUC approved protocol G-01-3 assigned to SMB. All procedures were also in compliance with the NRC “Guide for the Care and Use of Laboratory Animals”. Zebrafish embryos were obtained from natural crosses and staged according to Kimmel et al. [42]. WISH was performed as described [43]. For WISH on embryos older than 24 hpf, *N*-phenylthiourea (Sigma, Cat# 7629) was used to suppress pigment development. *mgat5a* probe was made using the following primers: probe-F: CAG AGG AGA ACC AAA GCG TGA TGGA; probe-R: GGA CCT CCA ACT GTG TTT TCC TGTC. 10 embryos per data point were used for WISH, and the representative images were shown. Capped mRNAs for *lefty1* and *squint* were synthesized using mMessage mMachine SP6 Transcription Kit (Ambion, Cat# AM1340M). Microinjections of these mRNA were performed in one-cell stage embryos. The injected embryos were sorted at 4 hpf, and only the healthy embryos were used for mock or SW treatment and then for morphological analysis at 24 hpf. For histological sectioning analysis, 5-day-old embryos from in situ hybridization analysis were embedded in paraffin, transversely sectioned at a thickness of 5 μm, and then counterstained by light nuclear fast red to better visualize the in situ staining.

### Analysis of *mgat5a* expression in neuromast cells

Three transgenic lines were used for analyzing *mgat5a* expression in neuromast cells: *mgat5a* ^*mn0157gt*^ [16], Et(*tkn1bp1*:*EGFP*) [18], and Et(ET20:*EGFP*) [19]. Embryos obtained from single or double transgenic line(s) were sorted based on the encoded fluorescent protein(s). The sorted embryos were used for mock or copper treatment at 5 dpf, mounted in 1 % low-melting agarose, and then imaged under a Meta-510 confocal microscope. For each data point, nine neuromasts from three embryos were examined and imaged, with the representative images shown.

### Mutation generation and characterization

*mgat5a* ^*mn0157gt*^ mutation was identified by a gene trap insertion [16]. *mgat5a* ^*mn0157gt*^ was genotyped using three primers, with mgat5a-F (CTC TAA CCA GTG ACC TTC TTG CTG) and mgat5a-R (CAA GGA ATT TCA AGT AAC GGT CAC) amplifying the WT allele of 350 bp, with Tol2N (AAT TAC TCA AGT ACT TTA CAC CTC T) and mgat5a-R amplifying the mutant allele of 196 bp. *mgat5a* ^*hg29*^ was generated by co-injecting 150 pg of Cas9 mRNA with 50 pg of each of two CRISPR single guide RNAs targeting exon 3 and exon 10 of *mgat5a*, as previously described [44]. The CRISPR target in exon 3 was GGG TTG ACT GGG TTT GGT CC; in exon 10, it was GGA CTC ATT CGG GAC GGA GC. The E3-E10 deletion mutation was detected using primers E3F (AAG ACT CTA GCT GTT CTT CTGG) and E10R (GAG TCT TGT GGC CTT TGG AC) with an amplicon size of 268 bp. E3F/E10R primers produced no amplicon for the WT allele. For knockdown efficiency analysis, RT-PCR was performed using 10 control and 10 mutant embryos at 3 dpf, with beta-actin as an internal reference. *mgat5a* transcript was detected by primers E9F (AAG GCG ATA AAG TGG TGG AGC TGA) and Ex12R (AGT GCC ATG GAC TGT GCC GTG AAC TTCC) for *mgat5a* ^*mn0157gt*^ and by primers E3F and Ex10R for *mgat5a* ^*hg29*^. Detailed information on the generation and detection of other CRISPR/Cas9 mutations are shown in Additional file 2: Table S1.

### Hair cell analysis

For hair cell development analysis, the embryos at 5 dpf were used for hair cell staining and counting. For hair cell regeneration analysis, the embryos at 5 dpf were exposed to 10 μM of copper sulfate (Sigma, Cat# 451657) for 2 h, allowed to recover for 48 h except when otherwise indicated, and then used for hair cell staining and counting. Hair cell staining by YO-PRO-1 (Thermo Fisher Scientific, Cat# Y3603) was done as previously described [18]. The stained embryos were oriented for lateral views in a 96-well plate for counting and imaging with a fluorescent microscope. Hair cells per neuromast presented in graphs were obtained by averaging the hair cell counts from four neuromast per embryos at the P1, P2, P4, and P5 positions [45] from multiple embryos. P2 neuromast was not counted, since it had a greater variation in the number of hair cells. For studying genotype and phenotype correlation, 24–32 embryos obtained from a single pair of heterozygote incross were analyzed and then genotyped. For studying the effect of chemical treatment, the number of wild-type embryos used for each data point was 10, except when otherwise indicated.

### Chemical treatment

The chemicals used in this study were as follows: SW (Sigma, Cat# S9263), DNJ (Sigma, Cat# D9305), and DAPT (Sigma, Cat# D5942). All chemical treatments were performed in a 6-well plate, with each well containing 5 ml of 1 × Holtfreter’s buffer [46] and fewer than 25 embryos. For measuring the effect on hair cell regeneration, WT embryos were ablated hair cells with 10 μM copper for 2 h at 5 dpf, treated with or without the chemical inhibitor for 48 h (except when otherwise indicated), and then counted hair cells as described. For measuring the effect on caudal fin regeneration, WT embryos at 3 dpf were anesthetized and amputated at the rear end of ventral pigment gap, treated without or with the indicated chemicals for 72 h, and then used for measurement of caudal fin area. The caudal fin area was measured by Image J software, using the anterior end of ventral pigment gap as a visual reference.

### Lateral line axon regeneration analysis

To evaluate the effect of swansonine on posterior lateral line (pLL) nerve regeneration, Tg (*neuroD*:*EGFP*;*mpeg1*:*mCherry*) double transgenic larvae were pre-incubated in E3 medium containing 50 μg/ml swansonine in 0.5 % DMSO for 15 h before neurectomy, from 57 to 72 hpf. As controls, we used larvae incubated only with 0.5 % DMSO. The 3 dpf pre-incubated larvae were anesthetized with 0.01 % tricaine and mounted in rectangular plates sealed with low melting point agarose (0.75 %). The agarose was dissolved in E3 medium containing DMSO or swansonine at the same concentration as the pre-treatment in order to avoid treatment interruption. Once the agarose was set, the embryos were neurectomized [47, 48] using a tungsten electrode connected to a power source under the following conditions: 1 pulse of 1.5 s of duration and 17 μA of current intensity. The pLL nerve was interrupted midway between the pLL ganglion and the L1 neuromast of the primary lateral line, approximately at the fourth somite. After neurectomy, larvae were dismounted and maintained in fresh 2 ml E3 medium containing swansonine or DMSO at 28 °C for 24 h post neurectomy (hpn). Larvae that displayed partial pLL nerve ablation or larvae with an intact pLL nerve were removed from the experiment. After 24 hpn, selected embryos were anesthetized and mounted in agarose plates in order to evaluate nerve regeneration. Using an epifluorescence microscope (Olympus, model IX81), we quantified nerve regrowth by recording the posteriormost somite reached by the nerve after 24 hpn in control and drug-treated larvae. The results were obtained from three independent experiments and a total of 45 control and 33 swansonine-treated larvae were analyzed. All data analysis was performed using Prism 5.0b (GraphPad Prism Software, Inc., USA).

### Statistical analysis

The statistics were performed using the Student *t* test (two tailed) except otherwise indicated. A difference was considered as significant when the *p* value was less than 0.05. Bar graphs showed the mean and the standard error of the mean (s.e.m.). All experiments shown were replicated at least two times and produced consistent results.

## Additional files

Additional file 1: Figure S1. CRISPR mutations of other genes in the *N*-glycosylation pathway. The pathway steps for CRISPR-mutated genes are shown. For the genes duplicated in zebrafish, including (*man1a*,*b*), (*man2a*,*b*), and (*mgat4a*,*b*,*c*), each of the replicated copy was mutated and analyzed for the regeneration individually. **Figure S2.** Inhibition of *N*-glycosylation shows no effect on the responsiveness to TGF-beta agonist Squint. (A) Classification of morphological phenotypes of WT embryos injected with *squint* mRNA. WT embryos were injected with 10 pg of *squint* mRNA at 1-cell stage, treated without or with 50 μg/ml SW from 4 to 24 hpf and then analyzed for morphological phenotypes. cI–cIII, class I–class III. cIV embryos were dead from over-involution at the time of analysis. (B) Percentage of embryos with different classes of phenotypes. The number of embryos analyzed is as indicated. The difference between the control and SW treated groups is not significant (n. s., *p* = 0.33). (PDF 612 kb)

Additional file 2: Table S1. CRISPR targets and mutation detection primers for the genes in *N*-glycosylation pathway. (XLS 46 kb)

## Abbreviations

Cas9: CRISPR-associated protein 9
CRISPR: Clustered regularly interspaced short palindromic repeats
DAPT: *N*-[(3, 5-Difluorophenyl) acetyl-L-alanyl-2-phenyl] glycine-1,1-dimethylethyl ester
DNJ: 1-Deoxynojirimycin
Dpa: Days post ablation
GFP: Green fluorescent protein
Indel: Insertion/deletion
Mgat5a: beta1,6-*N*-acetylglucosaminyl-transferase V
RFP: Red fluorescent protein
SW: Swainsonine
WISH: Whole-mount in situ hybridization
