# Supplementary figures and images for: Loss of Mgat5a-mediated N-glycosylation stimulates regeneration in zebrafish
Source: Cell Regen. 2016 Oct 20;5:3. doi: 10.1186/s13619-016-0031-5 (PMC5072312; doi:10.1186/s13619-016-0031-5)

Suppl. Fig. 1

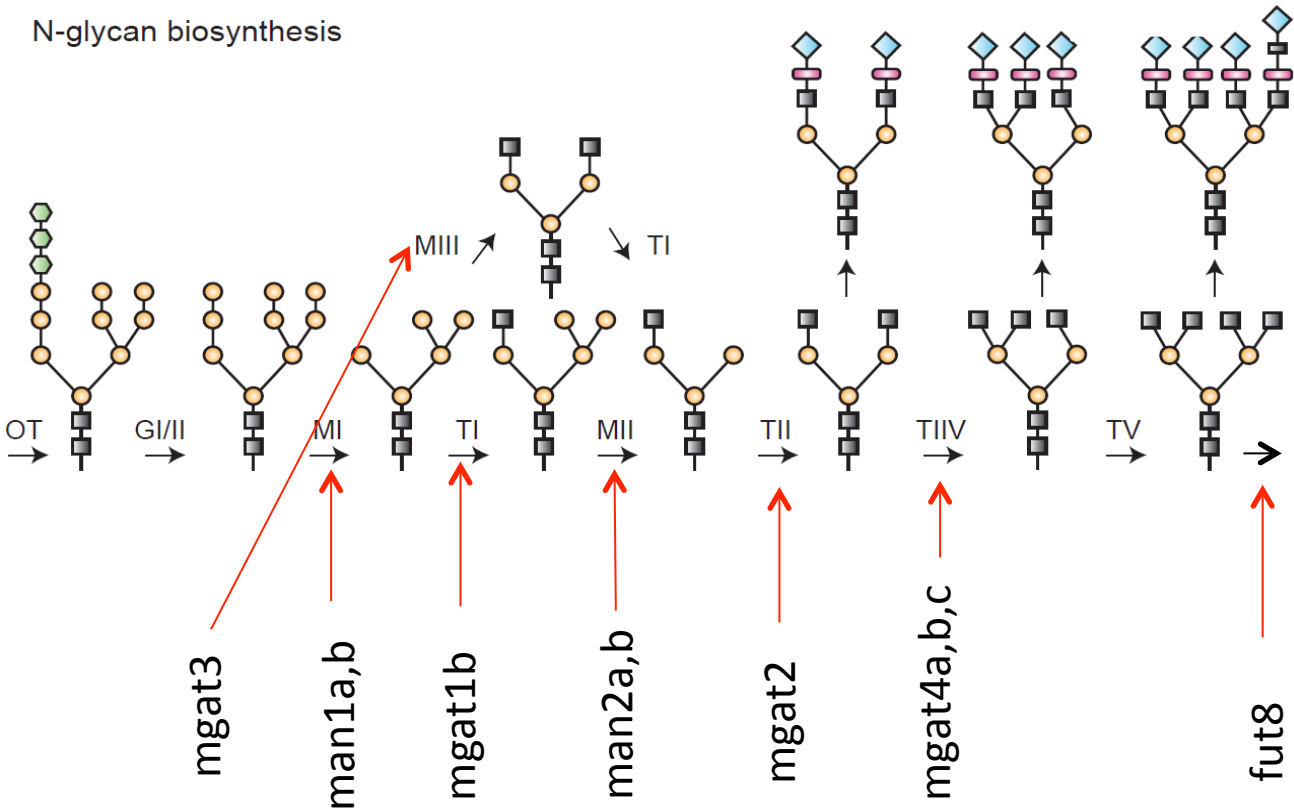

## Suppl. Fig. 2

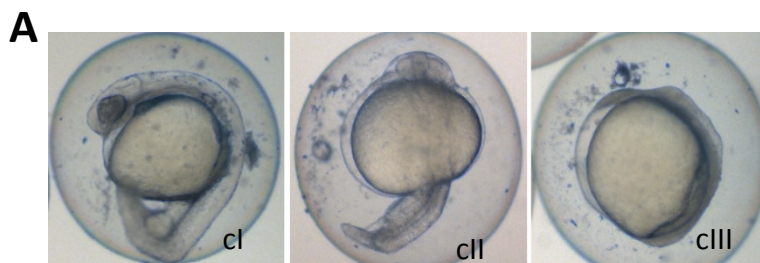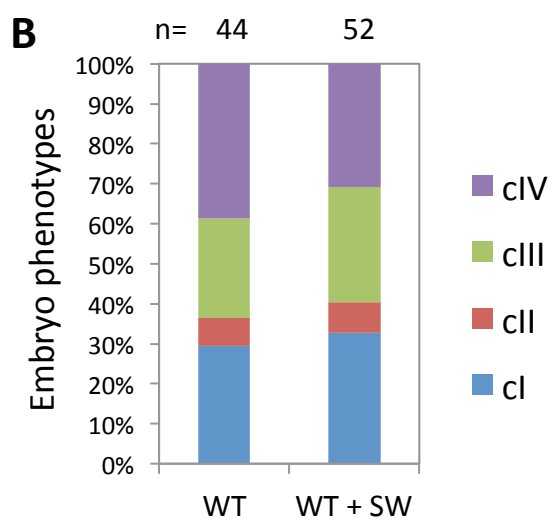

Supplement: Supplementary file 1 — CRISPR mutations of other genes in the N-glycosylation pathway. The pathway steps for CRISPR-mutated genes are shown. For the genes duplicated in zebrafish, including (man1a,b), (man2a,b), and (mgat4a,b,c), each of the replicated copy was mutated and analyzed for the regeneration individually. Figure S2. Inhibition of N-glycosylation shows no effect on the responsiveness to TGF-beta agonist Squint. (A) Classification of morphological phenotypes of WT embryos injected with squint mRNA. WT embryos were injected with 10 pg of squint mRNA at 1-cell stage, treated without or with 50 μg/ml SW from 4 to 24 hpf and then analyzed for morphological phenotypes. cI–cIII, class I–class III. cIV embryos were dead from over-involution at the time of analysis. (B) Percentage of embryos with different classes of phenotypes. The number of embryos analyzed is as indicated. The difference between the control and SW treated groups is not significant (n. s., p = 0.33). (PDF 612 kb) [file 13619_2016_31_MOESM1_ESM.pdf]
